# Dengue Fever in Mainland China, 2005–2020: A Descriptive Analysis of Dengue Cases and *Aedes* Data

**DOI:** 10.3390/ijerph19073910

**Published:** 2022-03-25

**Authors:** Yujuan Yue, Qiyong Liu, Xiaobo Liu, Ning Zhao, Wenwu Yin

**Affiliations:** 1 State Key Laboratory of Infectious Disease Prevention and Control, National Institute for Communicable Disease Control and Prevention, Chinese Center for Disease Control and Prevention, Beijing 102206, China; yueyujuan@icdc.cn (Y.Y.); liuqiyong@icdc.cn (Q.L.); liuxiaobo@icdc.cn (X.L.); zhaoning@icdc.cn (N.Z.); 2 Chinese Field Epidemiology Training Program, Chinese Center for Disease Control and Prevention, Beijing 102206, China; 3 Division of Infectious Disease Management, Chinese Center for Disease Control and Prevention, Beijing 102206, China

**Keywords:** dengue fever, *Aedes albopictus*, *Aedes aegypti*, time-series analyses, spatial occurrence frequency analyses, spatial diffusion analyses

## Abstract

Dengue fever occurs throughout mainland China, except in the Tibet Autonomous Region. During 2005–2020, there were 12,701 imported cases and 81,653 indigenous cases recorded. The indigenous cases were mainly clustered in Guangdong (74.0%) and Yunnan provinces (13.7%). Indigenous dengue fever is a seasonal illness in mainland China, manifesting predominantly in summer and autumn. Indigenous dengue fever cases tend to peak every 5years and have shown a substantial increase during the period 2005–2020. During the study period, indigenous dengue fever occurred more than ten times in each of the seven counties of Guangdong Province. Indigenous dengue fever has spread from low to high latitudes; that is, from the southwestern, southern, and southeastern areas to the central and northern regions, and from border ports and cities to rural areas. *Aedes aegypti* has become widespread in Yunnan Province but has diminished in Guangxi, Guangdong, and Hainan provinces in recent years. *Aedes albopictus* is distributed throughout mainland China, spanning 25 provinces and municipalities. To maintain effective public health prevention and control, it is important to monitor dengue occurrence, provide dengue classification guidance, and ensure sustainable vector management of *Aedes*.

## 1. Introduction

Dengue fever is a viral illness, mainly transmitted by *Aedes aegypti* and *Aedes albopictus*, and is one of the most prevalent mosquito-borne diseases in humans. There are four virus serotypes, namely Dengue I–IV. Dengue virus can cause dengue fever, dengue hemorrhagic fever, and, in severe cases, dengue shock syndrome [1]. The first recorded outbreak of dengue fever was in Jakarta, Indonesia, in 1779. Dengue fever is currently endemic in more than 100 countries, including Southeast Asia, the Americas, the Western Pacific, Africa, and the eastern Mediterranean region [2]. A study in 2013 estimated that 390 million people had dengue virus infections with 96 million cases annually worldwide, more than three times higher than the World Health Organization’s 2012 estimates [3].

Dengue fever was first reported in Xiamen City, Fujian Province, China, in 1873. Apart from a serious epidemic of dengue fever in China from 1940 to 1945, no further epidemic has been recorded for more than 30 years [1]. Dengue fever was prevalent in Foshan City, Guangdong Province, in 1978, with 22,122 cases reported [4]. There were large, localized outbreaks of dengue fever in Hainan Island in 1980 and 1986, with 454,664 and 118,987 cases reported, respectively, and there was a large outbreak of dengue fever in Foshan City, Guangdong Province, in 1995, with 6836 cases reported [5]. Dengue fever swept through Guangdong Province, China, in 2014, with approximately 47,000 cases [6], and in Guangdong and Yunnan provinces in 2019, with 12,908 cases reported [7]. From 1978 to 2014, 708,073 cases and 616 deaths were reported in mainland China [8]. Dengue fever outbreaks usually spread from the southern coastal areas, such as Guangdong and Hainan, towards the more northern and western areas, including Fujian, Zhejiang, and Yunnan. Shorter outbreak intervals have been recorded since the 1990s compared to before that time [9]. Most studies on the epidemiological characteristics of both imported and indigenous dengue fever in China have paid attention to spatial, temporal, and demographic distributions in short time series. However, demographic characteristics would be unlikely to change over a relatively short time. Furthermore, we note that dengue remains an imported disease in China [10]. Therefore, this study focused on the spatiotemporal dynamics of indigenous dengue fever in mainland China, especially spatial diffusion and spatial occurrence frequency. The objective was to provide scientific information for public health practitioners to formulate targeted strategic plans and implement effective public health prevention and control measures.

## 2. Materials and Methods

### 2.1. Data Collection

Dengue cases were defined based on three successive editions of criteria/guidelines for dengue diagnosis issued by the Chinese Ministry of Health in 2001, 2008, and 2018 (WS 216-2001, WS 216-2008, and WS 216-2018) [11,12,13].

Dengue fever, a vector-borne notifiable disease in China, is reported to the Chinese Centre for Disease Control and Prevention (China CDC) through the Chinese National Notifiable Infectious Disease Reporting Information System (CNNDS) (http://www.chinacdc.cn/, accessed on 30 June 2021). Dengue case reports include sex, age, occupation, national current address code, date of onset of illness, and remarks. Dengue case reports in mainland China from 1 January 2005 to 31December 2020 were obtained from CNNDS. *Aedes* data were obtained from the Chinese National Vector Surveillance System (CNVSS). CNVSScollected and recorded the previous active investigations of *Aedes* from the Chinese CDC, scientific works of literature of *Aedes*, as well as the routine monthly monitoring results of *Aedes* by county-, prefecture-, and province-level Centers for Disease Control and Prevention. Demographic data at the county level from the sixth population census (2010) were obtained from the National Bureau of Statistics of China (http://www.stats.gov.cn/, accessed on 30 June 2021). The vector data related to Chinese administrative divisions in 2017 were used for geographical mapping and were provided by CNNDS.

Ethics statement: No human or animal samples were included in the research presented in this article; therefore, ethical approval was not necessary for this research.

### 2.2. Data Processing

Dengue cases were divided into indigenous cases, imported cases, and other cases, according to the diagnostic criteria and principles of the management of dengue fever in China. Indigenous infections were classified as those in people who had not moved out of their local counties (current addresses) for 14 days before the onset of illness. Finally, indigenous cases occurred from May to December annually in this study. Imported cases were defined as those in people who had visited foreign countries or regions where dengue fever was prevalent, up to 14 days before the onset of illness. Our category of “other cases” included all those that we were unable to classify [14].

There are 31 provinces (or municipalities) comprised of 2922 counties in mainland China, with populations ranging from 7123 to 5,044,430, and geographic areas ranging from 5.4 to 197,346 square kilometers [15]. To perform spatial analysis, dengue cases were aggregated at the county level, and then geocoded and matched to county level administrative boundaries using ArcGIS, version 10.3 (ESRI Corp., Redlands, CA, USA). Dengue cases were also aggregated at the month level for follow-up analyses.

### 2.3. Data Analysis

Time-series analyses based on the line chart were conducted using IBM SPSS Statistics, version 24.0 (IBM Corp., Armonk, NY, USA). Spatial occurrence frequency is a measure of dengue fever occurrence in a county over a number of years. The value of each year is 0 or 1. To compare yearly patterns of indigenous cases, indigenous dengue cases from 2005 to 2020 were standardized, and R Statistical software, version 4.0.2 (R Foundation for Statistical Computing, Vienna, Austria) was used to produce heat maps and conduct statistical analyses. The heat map was a false-color image with a dendrogram added to the left side and to the top. Typically, a reordering of the rows and columns according to the means of indigenous cases within the restrictions imposed by the dendrogram was carried out. Spatial distribution analyses were conducted using ArcGIS, version 10.3. Spatial diffusion analyses of indigenous dengue cases from 2005 to 2020 were conducted using the global polynomial interpolation and contour in ArcGIS, version 10.3. Global polynomial interpolation fits a smooth surface that is defined by a polynomial in the input sample points. The result is a smooth surface that represents gradual trends in the surface over the area of interest. Contours are lines that connect locations of equal value in a raster dataset that represent continuous phenomena. When the power of global polynomial interpolation was set as 3 in this study, the trend surface of dengue fever had the smallest root mean square. Contours indicating the invasive years of indigenous dengue cases were drawn based on the trend surface. The slope of the trend surface represents the reciprocal spread speed of indigenous dengue cases from 2005 to 2020.

## 3. Results

### 3.1. Dengue Fever in Mainland China, 2005–2020

**Figure 1:**
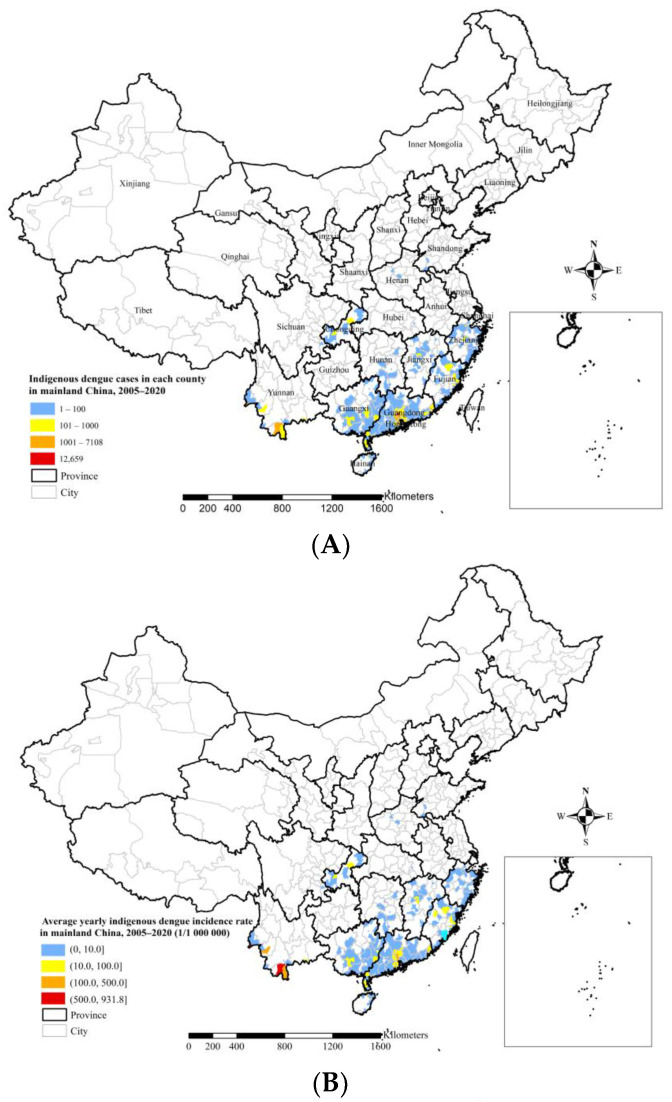

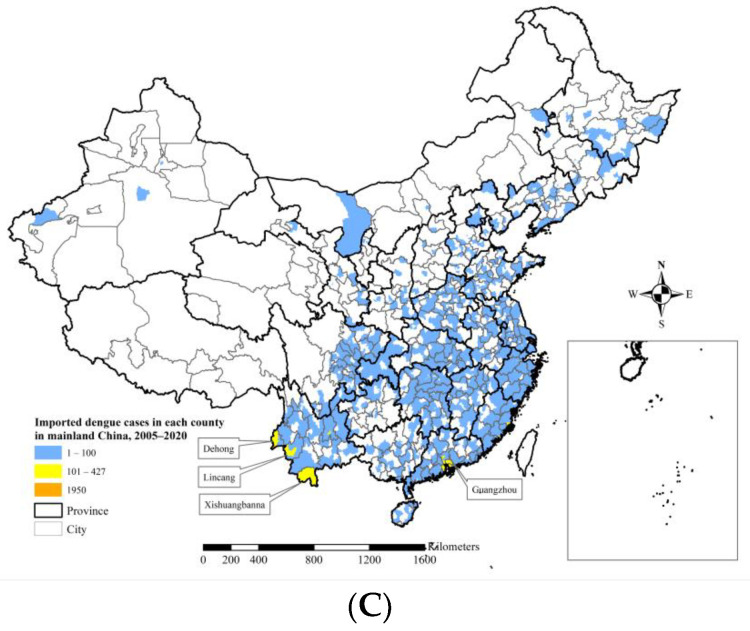
Spatial distribution of dengue fever in mainland China during 2005–2020. (**A**) Indigenous cases. (**B**) Indigenous incidence rate. (**C**) Imported cases.

There were 81,653 indigenous dengue cases reported in mainland China from 2005 to 2020, across 345 counties in 14 provinces and municipalities (Figure 1A and Table 1). There were periodic peaks of 1007, 46,034, and 15,378 indigenous cases in 2006, 2014, and 2019, respectively. Compared with the number of indigenous cases in adjacent years, there was also a small peak in 2019. The overall trend showed a marked increase in both the number of cases and affected counties. The largest indigenous cases at the county level in 2005–2020, 12,659 cases, were distributed in Baiyun County, Guangzhou City, and Guangdong Province. Dengue fever broke out in Guangzhou in 2014, with 11,834 indigenous cases reported in Baiyun County. Over this period, indigenous cases were largely distributed in the southeastern provinces of Guangdong, Yunnan, Guangxi, and Fujian, accounting for 74.0%, 13.7%, 3.3%, and 2.8% of the total cases, respectively. However, dengue fever was also detected in Chongqing Municipality for the first time in 2019, with its indigenous cases accounting for 1.5% of the total.

The top two average annual indigenous incidence rates at the county level in 2005–2020 were 931.8 and 854.4 cases per 1,000,000 people in Jinghong City, Xishuangbanna Dai Autonomous Prefecture, and Ruili City, Dehong Dai Jingpo Autonomous Prefecture, Yunnan Province, respectively. Yunnan and Guangdong provinces both recorded average annual indigenous incidence rates of more than 100 cases per 1,000,000 people at the county level (Figure 1B).

There were 12,701 imported cases in China from 2005 to 2020, which occurred across 1400 counties in 30 provinces and municipalities (Figure 1C and Table 1). There was a large-scale increase in the number of imported cases during the time period, reaching 5813 cases in 2019, which accounted for 45.8% of total imported cases in just that one year. There was also a significant increase in the number of counties affected by imported dengue fever, reaching 1090 counties in 2019, with that year accounting for 77.9% of the total affected counties with imported dengue fever for 2005–2020. There was one imported case in Xining City, Qinghai Province in 2009, but its national current address code was wrong, and therefore, this is not marked in Figure 1C. The largest number of imported cases at the county level, 1950 cases, was found in Ruili City, Dehong Dai Jingpo Autonomous Prefecture, Yunnan Province. Imported cases were mainly distributed in Yunnan, Guangdong, Fujian, and Zhejiang provinces, accounting for 36.1%, 19.5%, 8.9%, and 8.0% of the total imported cases, respectively.

### 3.2. Indigenous Dengue Fever inMainland China, 2005–2020

#### 3.2.1. Time-Series Analyses

**Figure 2:**
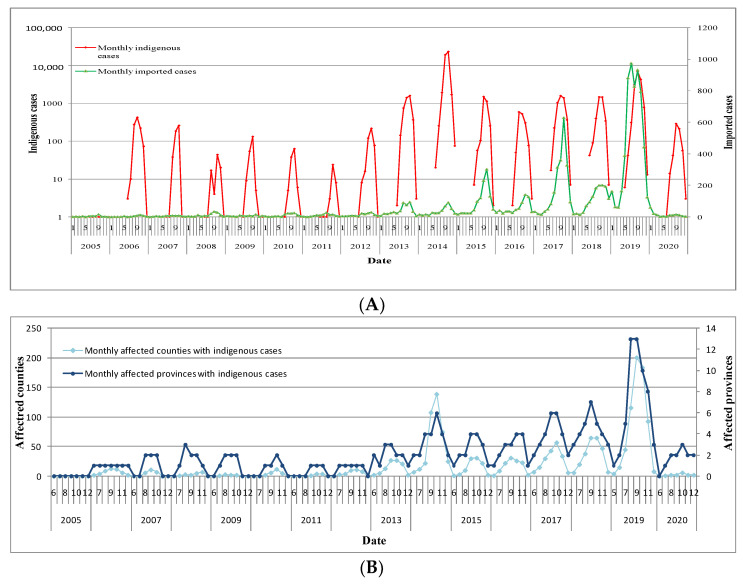
Time-series analyses of dengue cases from 2005 to 2020. (**A**) Monthly imported dengue cases from January to December 2005–2020, and monthly indigenous dengue cases from June to December 2005–2020, and in May 2019. (**B**) Monthly affected counties and provinces with indigenous cases from June to December 2005–2020, and in May 2019.

No indigenous cases were recorded in 2005.Large dengue outbreaks occurred in 2014 and 2019, with 46,034 and 15,378 indigenous cases distributed in 160 and 266 counties, respectively. Annually, there was a seasonal trend of peaks in the number of cases occurring from July to November. The peak number of cases in the time period occurred in September and October 2014, with 18,980 and 23,105 indigenous cases in 108 and 139 affected counties of 4 and 6 provinces, respectively, and in September 2019, with 6901 indigenous cases in 201 affected counties of 13 provinces. Indigenous cases occurred from June to December before 2019 but occurred in May 2019.Imported dengue fever can theoretically manifest throughout the year; however, peaks of imported dengue cases generally occurred slightly later than, or at the same time as indigenous dengue case peaks (Figure 2).

#### 3.2.2. Spatial Occurrence Frequency Analyses

**Figure 3:**
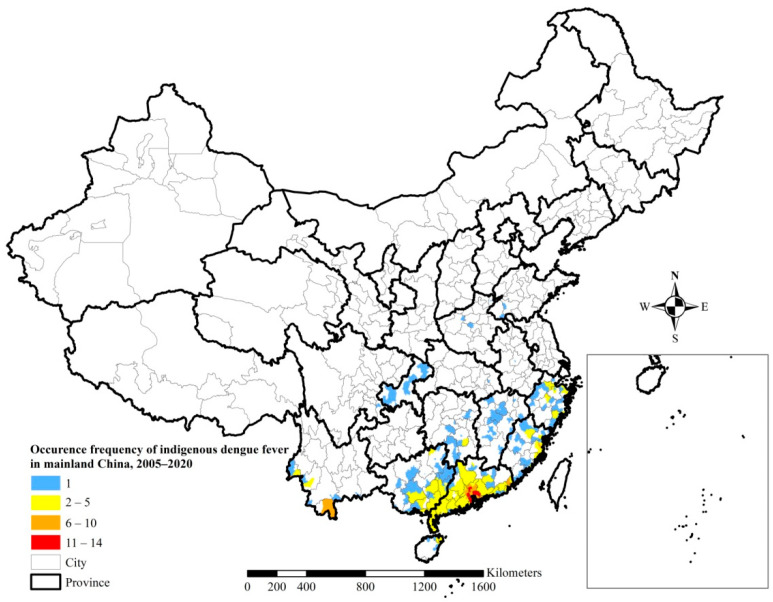
Occurrence frequencies of indigenous dengue fever in mainland China, 2005–2020.

Seven counties in Guangdong Province (four in Guangzhou City, and one each in Foshan City, Dongguan City, and Zhongshan City), recorded more than ten occurrences of indigenous dengue fever each from 2005 to 2020, while twenty-one counties in Yunnan, Guangdong, and Fujian provinces recorded between six and ten occurrences of indigenous dengue fever each (Figure 3).

#### 3.2.3. Heat Map and Spatial Diffusion Analyses

**Figure 4:**
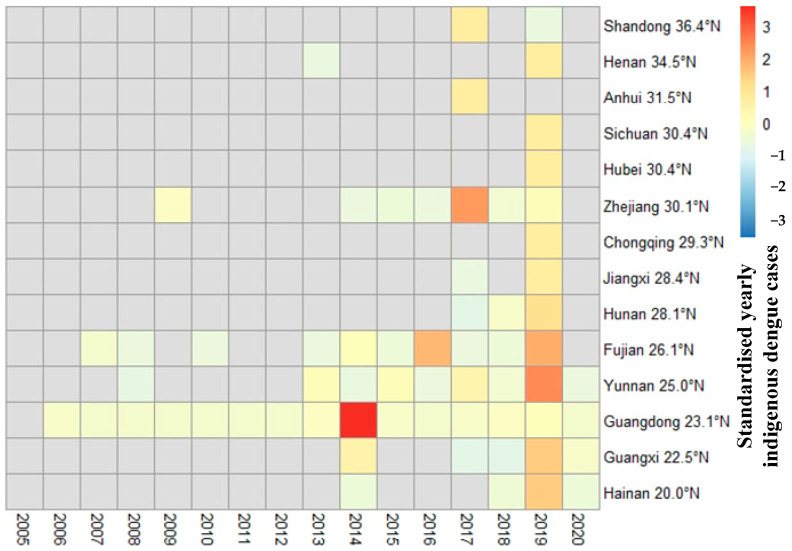
Heat map of indigenous dengue cases in mainland China in 2005–2020.

Indigenous dengue fever cases were largely concentrated in locations at low latitudes (Guangdong, Yunnan, and Fujian provinces), but it was also detected at relatively high latitudes (Henan and Shandong provinces), Figure 4. Indigenous dengue fever has spread considerably since 2014—only 5provinces were affected by indigenous dengue fever before 2014, but by 2020, there were 14 affected provinces; and 3 new jurisdictions (Chongqing Municipality, Sichuan Province, and Hubei Province) recorded cases in 2019.

**Figure 5:**
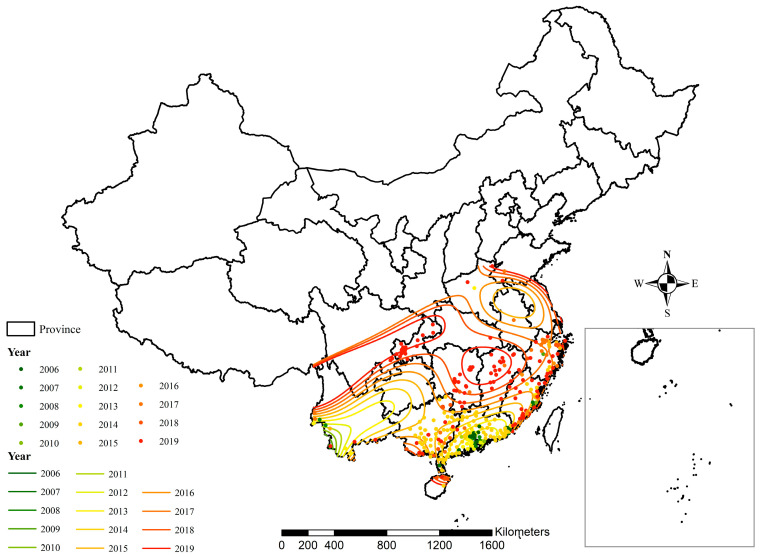
Spatial diffusion of indigenous dengue fever in mainland China, 2005–2019.

Figure 5 illustrates how indigenous dengue fever has spread from the southwestern, southern, and southeastern regions, towards the central and northern regions of mainland China, from 2005 to 2020.

### 3.3. Analyses of Aedes aegypti and Aedes albopictus in Mainland China

**Figure 6:**
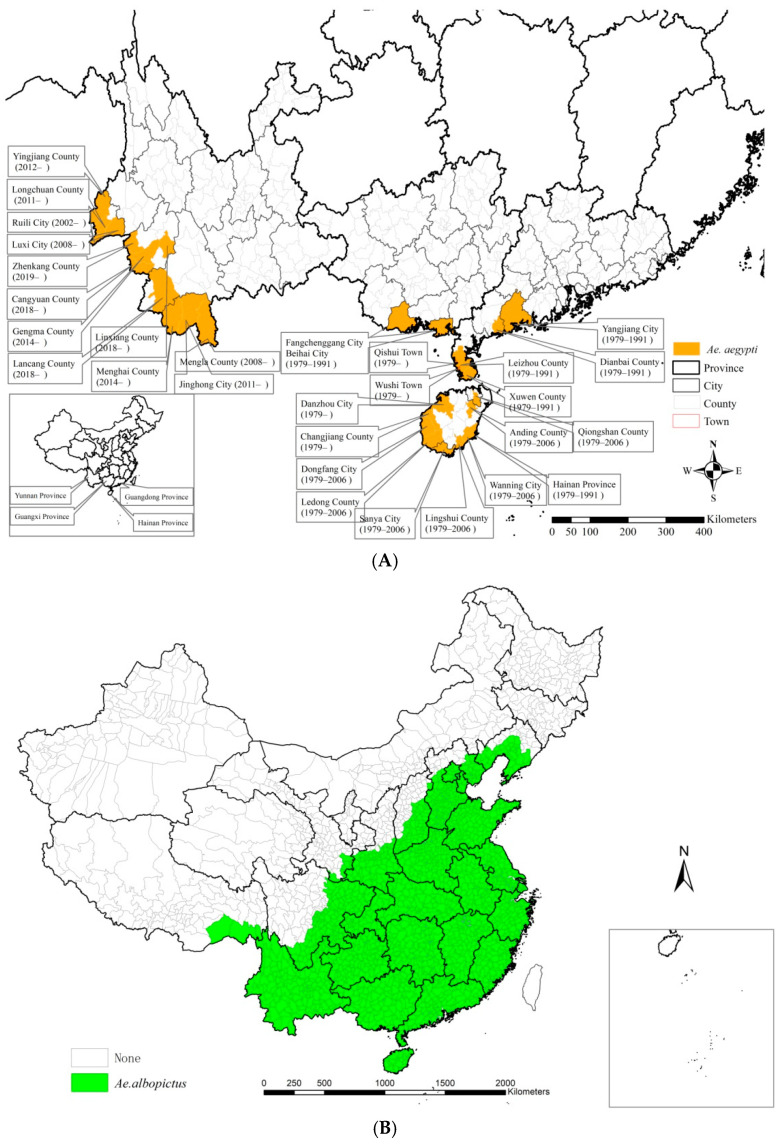
Spatial distributions of *Aedes* in mainland China. (**A**) *Aedes aegypti*. (**B**) *Aedes albopictus*.

From 1991 to the early 2000s, the distribution of *Aedes aegypti* was concentrated in the Guangdong, Hainan, and Guanxi provinces in southern China. Its presence has contracted in these areas since then while being increasingly detected in more recent years in 12 border counties in Yunnan Province far to the west (Figure 6A). *Aedes albopictus* is widely distributed in 25 provinces and municipalities to the southeast of a line that runs through the provinces of Liaoning, Hebei, Shanxi, Shaanxi, Gansu, Sichuan, and Tibet (Figure 6B). *Aedes aegypti* and *Aedes albopictus* were both distributed in Yunnan, Guangxi, Guangdong, and Hainan provinces along the southwestern and southern coastal areas of China.

## 4. Discussion

Two national databases, CNNDS and CNVSS, which are very important, authoritative, and comprehensive, were used to determine the epidemic status of dengue fever in mainland China from 2005 to 2020. The results of this data analysis, therefore, provide a sound scientific repository of information for use in dengue fever prevention and control in China.

We found that 81,653 indigenous dengue cases and 12,701 imported cases were reported from 2005 to 2020 across 345 counties in 14 provinces and municipalities, and 1400 counties in 30 provinces and municipalities, respectively (Table 1). Except for the Tibet Autonomous Region, dengue cases have occurred throughout China. There were only 13 deaths in the 15 years from 2005 to 2020, namely 1 in 2005, 6 in 2014, 2 in 2017, 1in 2018, and 3 in 2019. Most indigenous cases were clustered in Guangdong (74.0%) and Yunnan provinces (13.7%). Counties with high occurrence frequencies of indigenous dengue fever are mainly located in Guangdong (Figure 3). Therefore, Guangdong and Yunnan are the provinces with the most cases of dengue fever, which is attributed to the fact that Yunnan is adjacent to Myanmar which has widespread dengue fever, and Guangdong is a highly populated and developed province along the southern coast [16,17,18,19,20,21,22]. Over the time period studied, there has been an increase in imported cases in Yunnan, and the sources of imported cases in Guangdong are reportedly more extensive [14]. *Aedes aegypti* and *Aedes albopictus* are the main vectors for dengue fever transmission [2,3]. *Aedes aegypti* can spread dengue fever more effectively. Several outbreaks of dengue fever in Chinese history were caused by *Aedes aegypti* [4]. The distribution of *Aedes aegypti* has increased in Yunnan Province but has contracted in Guangxi, Guangdong, and Hainan provinces in recent years [5,23]. *Aedes albopictus* was distributed below a northeast-southwest line throughout southeastern China, spanning 25 provinces and municipalities (Figure 6) [24]. Dengue serotypes in China were mainly DV II and DV III before 1990, DVI and DVII during 1990–2000, and DVI after 2000. Dengue virus serotypes I–IV are all currently prevalent in China [1,5,9,25].

Dengue fever is directly related to *Aedes*. The epidemic situation of dengue fever is closely related to the prevention and control measures and efforts of dengue fever and *Aedes*, such as mosquito elimination and the establishment of hygienic cities. In response to the outbreak of dengue fever, institutions of disease prevention and control at all levels under the leadership of the government would increase efforts to invest human and material resources in dengue fever prevention and control. In response to the large dengue outbreak in 2014, Guangzhou City invested nearly 200 million RMB in mosquito control (http://news.cnr.cn/native/gd/20150116/t20150116_517448069.shtml, accessed on 30 May 2021). After a large-scale mosquito killing, the restoration of mosquito ecology needs a certain time. In addition, institutions of disease prevention and control at all levels have routine monitoring and control of mosquitoes. These may have been some of the reasons why dengue fever broke out every few years in China. Indigenous dengue fever has spread from low to high latitudes, from the southern areas to the central and northern regions (Figure 4 and Figure 5), and from border ports and cities to rural areas [26,27]. The growth and spread of dengue fever closely relate to social and economic development, climate change globalization, tourism, commerce and trade, migrant workers, and increased urbanization [28,29,30,31,32]. Dengue outbreaks caused by imported dengue cases had the characteristics of sudden, fierce, and rapid transmission [33]. More than half of China has *Aedes* vectors of dengue transmission. Therefore, with the factors of climate change, globalization, urbanization, population migration, and the spatial distribution of *Aedes*, it is possible to cause a spatial diffusion and pandemic of dengue fever in China.

Imported dengue cases potentially occur throughout the year and indigenous dengue outbreaks have sometimes coincided with imported dengue outbreaks (Figure 2). Affected by COVID-19 lockdowns, imported and indigenous cases were both very minimal in 2020. However, it is expected that when borders reopen, dengue will remain an imported disease in mainland China [10,34]. Indigenous dengue fever has clear seasonal characteristics, with caseloads peaking in summer and autumn annually, as is also true in neighboring tropical countries [35,36,37,38]. Indigenous dengue fever can occur in areas where the vectors *Aedes aegypti* and *Aedes albopictus* are distributed. However, from the perspective of the occurrence frequency of indigenous cases, the distribution of dengue fever and *Aedes*, dengue risk in the southwestern, southern, and southeastern border ports and cities of China was higher than that in rural areas, and in the distribution areas of *Aedes aegypti* than of *Aedes albopictus*. The risk level of contracting dengue fever, therefore, varies in different locations. Classification guidance for dengue fever risk should be implemented, according to the Technical Guide for Dengue Fever Control (www.chinacdc.cn, accessed on 25 August 2021). *Aedes* control can refer to sustainable vector management [39]. There are several effective public health measures used to minimize the risk of dengue fever, including controlling vectors, such as *Aedes aegypti* and *Aedes albopictus*, community participation, health education, and environmental measures [40,41].

A limitation of the study isa lack of information regarding some indigenous dengue records in the CNNDS database. Records were categorized mainly according to the remarks on dengue case reports, but some were recorded ambiguously or left blank. Therefore, we acknowledge that a few cases that were likely indigenous were omitted from this study.

## 5. Conclusions

Currently, dengue fever is widespread and a considerable burden on public health and the economy in China. Guangdong and Yunnan, where dengue fever occurs frequently, reported 87.7% of China’s indigenous cases between 2005 and 2020. Indigenous dengue fever has spread from the southwestern, southern, and southeastern regions towards the central and northern regions in recent years. The distribution of *Aedes*, the vector of dengue fever, spans 25 provinces and municipalities across a wide area of southeastern China. The rapidly rising number of cases and geographical spread means that there is an urgent need to implement better public health measures against dengue fever. Understanding the current situation of dengue fever in China, including the distribution and changing characteristics of *Aedes* and virus serotypes, is conducive to providing scientific suggestions for its effective prevention and control.

## Figures and Tables

**Table 1:** Comparison of indigenous and imported dengue cases in mainland China, 2005–2020.

Year	Dengue Cases		Counties with Dengue Fever	
	Indigenous	Imported	Indigenous	Imported
2005	0	45	0	33
2006	1007	46	15	33
2007	481	56	13	37
2008	86	134	11	62
2009	200	73	5	51
2010	112	119	14	64
2011	35	113	6	73
2012	438	149	14	86
2013	4263	460	36	182
2014	46,034	399	160	126
2015	3044	1083	44	264
2016	1549	675	41	211
2017	4609	2112	76	313
2018	3801	1266	100	457
2019	15,378	5813	266	1090
2020	616	158	7	101
In all	81,653	12,701	345	1400

## Data Availability

Not applicable.
